# The bending rigidity of the red blood cell cytoplasmic membrane

**DOI:** 10.1371/journal.pone.0269619

**Published:** 2022-08-01

**Authors:** Sebastian Himbert, Angelo D’Alessandro, Syed M. Qadri, Michael J. Majcher, Todd Hoare, William P. Sheffield, Michihiro Nagao, John F. Nagle, Maikel C. Rheinstädter

**Affiliations:** 1 Department of Physics and Astronomy, McMaster University, Hamilton, ON, Canada; 2 Origins Institute, McMaster University, Hamilton, ON, Canada; 3 Department of Pathology and Cell Biology, Columbia University Vagelos College of Physicians and Surgeons and New York-Presbyterian Hospital, New York, New York, United States of America; 4 University of Colorado Denver-Anschutz Medical Campus, Aurora, Colorado, United States of America; 5 Faculty of Health Sciences, Ontario Tech University, Oshawa, ON, Canada; 6 Department of Chemical Engineering, McMaster University, Hamilton, ON, Canada; 7 Department of Pathology and Molecular Medicine, McMaster University, Hamilton, ON, Canada; 8 Centre for Innovation, Canadian Blood Services, Hamilton, ON, Canada; 9 Center for Neutron Research, National Institute of Standards and Technology, Gaithersburg, MD, United States of America; 10 Department of Materials Science and Engineering, University of Maryland, College Park, MD, United States of America; 11 Department of Physics and Astronomy, University of Delaware, Newark, DE, United States of America; 12 Department of Physics, Carnegie Mellon University, Pittsburgh, PA, United States of America; Peking University, CHINA

## Abstract

An important mechanical property of cells is the membrane bending modulus, *κ*. In the case of red blood cells (RBCs) there is a composite membrane consisting of a cytoplasmic membrane and an underlying spectrin network. Literature values of *κ* are puzzling, as they are reported over a wide range, from 5 k_B_T to 230 k_B_T. To disentangle the contribution of the cytoplasmic membrane from the spectrin network, we investigated the bending of red blood cell cytoplasmic membranes (RBC_*cm*_) in the absence of spectrin and adenosine triphosphate (ATP). We used a combination of X-ray diffuse scattering (XDS), neutron spin-echo (NSE) spectrometry and Molecular Dynamics (MD) simulations. Our results indicate values of *κ* of order 4 k_B_T to 6 k_B_T, relatively small compared to literature values for most single component lipid bilayers. We suggest two ways this relative softness might confer biological advantage.

## Introduction

Cellular functions, such as mobility, division and vesicle trafficking, are intrinsically related to a cell’s ability to comply to deformation [1–3]. In the case of red blood cells (RBCs) that have no internal structure, this ability depends upon their two-dimensional “shell”, which consists of a spectrin network tethered to a cytoplasmic membrane.

A suite of techniques has been used to study cell elasticity. Mechanical properties on cellular length scales were measured by micropipette aspiration [4], while atomic force microscopy (AFM) [5] probes elastic behavior on the nanoscale. Cell stiffness is also studied indirectly by spectral analysis of flickering of cells under a microscope [6–8], as well as optical interferometric techniques [9, 10].

A particularly appropriate measure of elasticity is the bending modulus *κ*, which gives the energy required to bend away from the resting state. Table 1 shows values for the bending modulus *κ* of RBCs that have been reported over the years, ranging from 5 k_B_T to 230 k_B_T [4, 6–9, 11, 12]. A reasonable hypothesis for this disparity is that the bending modulus depends on the length scale of the measurements. On length scales smaller than the mesh size of the spectrin network of ≈80 nm, the average bending modulus could be due mostly to the cytoplasmic membrane, while the spectrin network would add a contribution at longer length scales [13]. When measuring RBC elasticity on small length scales, values for *κ* of 5 k_B_T [7] and 7 k_B_T [9] have been reported in contrast to the much larger values for length scales of the entire RBC [4, 8, 11, 12]. According to the above hypothesis, this would imply a substantial bending modulus for the spectrin network. It may be noted, however, this is inconsistent with a report that the bending modulus of the spectrin network is very small [14].

**Table 1:** A summary of values reported for the bending rigidity, *κ*, of discocytic red blood cells from the literature and our RBC_*cm*_ membranes. (Errors are expressed as standard deviations).

Technique	*κ* (k_B_T)	Lengthscale (*μ*m)	Reference
Chronological Literature	RBC		
Flickering Analysis	3–9	>0.6	[6]
Micropipette Aspiration Buckling	43	>7	[4]
Reflection Interference Microscopy	5±1.5	>0.25	[7]
Reflection Interference Microscopy	97±37	>2	[8]
Diffraction Phase Microscopy	16±0.3	>0.1	[10]
Reanalysis of [10]	14,25	>0.1	[13]
Flickering Analysis	210	>0.7	[11]
Optical Tweezer	68±0.68	>7	[12]
Flickering Analysis	67±13	>1.5	[30]
Diffraction Phase Microscopy	7±3	>0.1	[9]
Diffraction Phase Microscopy	5±2	>0.1	[31]
This paper	RBC_*cm*_		
Diffuse X-ray Scattering	2–6	$\}<0.08$	
Neutron Spin Echo	4–7		
Molecular Dynamics	4		

Supposing that the bending modulus of the cytoplasmic membrane is only about 5 k_B_T, it is noteworthy that this *κ* is significantly smaller than bending rigidities reported for single component lipid bilayers of similar thickness that have values of *κ* typically between 15 k_B_T and 50 k_B_T [15–28]. It is further intriguing that the cytoplasmic membrane contains considerable cholesterol which is conventionally thought to stiffen lipid membranes, although that depends on the lipid [19].

Here we measure the bending rigidity of the RBC’s cytoplasmic membrane to clearly separate the elastic contribution of the membrane from that of the spectrin network. We will call these RBC_*cm*_. Our samples also have no ATP (adenosine triphosphate), which has sometimes been reported to affect membrane fluctuations [12, 19], but sometimes not [11]. Solid-supported multi-lamellar RBC_*cm*_ stacks were prepared for measurements of X-ray diffuse scattering (XDS), and unilamellar RBC liposomes were prepared for neutron spin-echo (NSE) spectroscopy. We also performed coarse grained Molecular Dynamics (MD) simulations of multi-component membranes that essentially matched the lipid composition of the RBC_*cm*_ in the experiments. Table 1 shows our values of *κ* to facilitate comparison with literature values.

## Materials & methods

This research was approved by the Hamilton Integrated Research Ethics Board (HIREB) under approval number 1354-T. Informed consent was obtained from all blood donors by signing a written consent form. The authors confirm that all methods were performed in accordance with the relevant guidelines and regulations. Certain trade names and company products are identified in order to specify adequately the experimental procedure. In no case does such identification imply recommendation or endorsement by the National Institute of Standards and Technology (NIST), nor does it imply that the products are necessarily the best for the purpose.

### Preparation of RBC liposomes

10 ml of blood samples were collected from volunteers in heparanized blood collection tubes. RBC liposomes were then prepared from all samples following a previously published protocol [32, 33]. Briefly: The blood was washed twice and the RBCs were isolated by successive centrifugation and replacing the supernatant with phosphate saline buffer (PBS). The cells were exposed to osmotic stress by mixing hematocrit with lysis buffer (3% PBS buffer, pH 8) at a volume fraction of 5%. The lysis buffer was pre-chilled to ≈4°C and the reaction tube was immediately stored on ice to prevent a fast re-closing of the ruptured cells. Hemoglobin and other cellular compartments were removed through multiple washing steps, as demonstrated in [32]. The protocol results in a white pellet containing empty RBC vesicles, commonly known as RBC ghosts.

These RBC ghosts were suspended in heavy water (D_2_O) in the case of the NSE experiment: the supernatant was removed from the pellet and the tube was refilled with D_2_O. The sample was centrifuged at 20,000 g for 20 minutes and the resulting supernatant was subsequently replaced with D_2_O. This step was repeated twice. This buffer exchange was omitted when preparing the samples for the XDS experiment.

The resulting ghost solution was then tip sonicated 20 times for 5 s each at a power of 100 W. The reaction tube was placed on ice during sonication to prevent the sample from overheating. Afterwards, the tube was centrifuged for 15 min at ≈20,000 g. This additional centrifugation step was found to be an efficient method for removing remaining undesired structures from the solution: The supernatant consists of a solution of large unilamellar vesicles (LUV, diameter: 199 nm, PID = 0.1) while any larger structures sediment into a pellet. This supernatant has an approximate concentration of ≈14 mg/ml [33] and will be hereafter referred to as the *membrane solution*.

Multi-lamellar, solid supported membranes were prepared for the X-ray experiments. Membranes from the *membrane solution* were applied onto single-side polished silicon wafers. 100 mm diameter, 300 *μ*m thick silicon wafers were pre-cut into 10 × 10 mm^2^ chips. The wafers were treated with a solution of 15 ml sulfuric acid and 5 ml hydrogen peroxide (Piranha solution) resulting in a hydrophilic surface. Each wafer was then thoroughly rinsed with ≈50 ml of ultra pure water (18.2 M*Ω*⋅cm) and placed on a hot plate (37°C) in a 3-dimensional orbital shaker. 100 *μ*l of the *membrane solution* was pipetted slowly onto the wafer. The sample was covered with a tilted lid of a petri dish and to allow the membrane solution to slowly dry within ≈12 h. The dried wafers were further incubated prior to the experiment at 97% relative humidity and 37°C for 72 h by placing the samples in a sealed container with a saturated K_2_SO_4_ solution. This allows the membranes to assemble into an oriented multilamellar structure. Assuming an average area per lipid of 0.5 nm and an average molar mass per lipid of 700 g/mol permits determining the average mass per leaflet to be 400 *μ*g. A total membrane mass of 1.4 g/wafer thus results in of roughly 3,000 stacked membranes in a sample 18 *μm* thick.

The *membrane solution* suspended in D_2_O were used for the neutron spin-echo experiments to create a strong scattering contrast between the protonated RBC_*cm*_ and the surrounding solution. The liposome solution was brought to a final concentration of 20 mg/ml. First the sample was centrifuged at 20,000 g for 20 minutes and the supernatant replaced by D_2_O. This process was repeated twice. ≈6 ml of this solution was filled in custom made sample holders provided by the NIST Center for Neutron Research (NCNR). All samples were sealed prior to the shipment to the National Institute of Standards and Technology (NIST) in Gaithersburg, MD, USA. The vesicle diameter was measured using dynamic light scattering (DLS) prior to shipment and a diameter of 199 nm (polydispersity index = 0.1) was determined. Small Angle Neutron Scattering (SANS) experiments were performed at NIST simultaniously to the NSE experiments. The DLS results explain the SANS spectrum that was measured prior to the NSE experiment and is shown in S2 Fig in S1 File. Importantly, the SANS data showed no multilamellar peak confirming that the vesicles have a unilamellar structure.

We note that we can not fully exclude effects of the preparation protocol on the RBC_*cm*_s bending rigdity. An increased bending rigidity in stomatocytes has been reported previously [9] and it was speculated that this originated in changes of the lipid bilayer’s composition. Our simulations are critical as they provide a deeper insight into the effects of the lipid composition on the RBC_*cm*_ bending rigidity. The bending modulus of the asymmetric membrane was very close to values determined on symmetrized membranes models. Even the complete loss of polyunsaturated lipids or cholesterol increased the bending modulus to only 13 k_*B*_T. This demonstrates that even such extreme changes would result in bending moduli that are still significantly lower than values measured on synthetic lipid bilayers.

### X-ray diffraction

X-ray scattering experiments were performed using a rotating anode instrument equipped with a Rigaku HyPix-3000 2-dimensional semiconductor detector. Details of the experimental setup and protocol can be found in the *Supplementary Material*. The membrane bending modulus *κ* and the membrane interaction modulus *B* were determined from measurements of the diffuse scattering when the membranes were well-hydrated from water vapor close to 100% relative humidity. The analysis was similar to previous studies [16–20, 34], although the different experimental setup required a modification that is detailed in *Supplementary Material*. All measurements were conducted at 37°C. Basically, the *q*_||_ dependence of the intensity *I*(*q*_*z*_, *q*_||_) is proportional to a constant times the so-called structure or interference factor *S*(*q*_*z*_, *q*_||_). (The constant is related to the electron density profile which is not of concern in this paper and is a simple linear fitting parameter for each *q*_*z*_.) For obtaining moduli, the focus is the structure factor [34],

$$\begin{array}{c} \begin{array}{c} S\left(q_{z},q_{\left|\right|}\right)=\sum_{n=-\infty }^{n=\infty }H_{z}\left(nd,L_{z},\sigma_{z}\right)\text{cos}\left(q_{z}nd\right)\\ \times \int_{0}^{\infty }rdrH_{r}\left(r,L_{r},\sigma_{r}\right)J_{0}\text{exp}\left(-q_{z}^{2}\delta u_{n}\left(r\right)/2\right), \end{array} \end{array}$$ (1)

where *d* is the average repeat spacing of the membranes in the stack, *J*_0_ is the zero order Bessel function [34], *H*_*z*_(*z*, *L*_*z*_, *σ*_*z*_) and *H*_*r*_(*r*, *L*_*r*_, *σ*_*r*_) account for finite domain sizes within the sample; *L*_*r*_ and *L*_*z*_ are the average domain sizes with variances *σ*_*r*_ and *σ*_*z*_ in the lateral and out-of-plane directions [34]. The height-height pair correlation function *δu*_*n*_(*r*) follows from Eq (9) that defines *κ* and *B*

$$\begin{array}{c} \delta u_{n}\left(r\right)=\frac{2\eta_{c}}{q_{1}^{2}}\int_{0}^{\infty }dx\frac{1-J_{0}\left(r/\xi \sqrt{2x}\right){\left(\sqrt{1+x^{2}}-x\right)}^{2n}}{x\sqrt{1+x^{2}}} \end{array}$$ (2)

in which the Caillé *η*_*c*_ parameter [35] and an in-plane correlation length *ξ* are related to the bending modulus *κ* and the membrane interaction modulus *B* by

$$\begin{array}{c} \eta_{c}=\frac{k_{B}Tq_{1}^{2}}{8\pi \sqrt{B\kappa }}\;\text{and}\;\xi^{4}=\frac{\kappa }{B}. \end{array}$$ (3)

**Fig 1:**
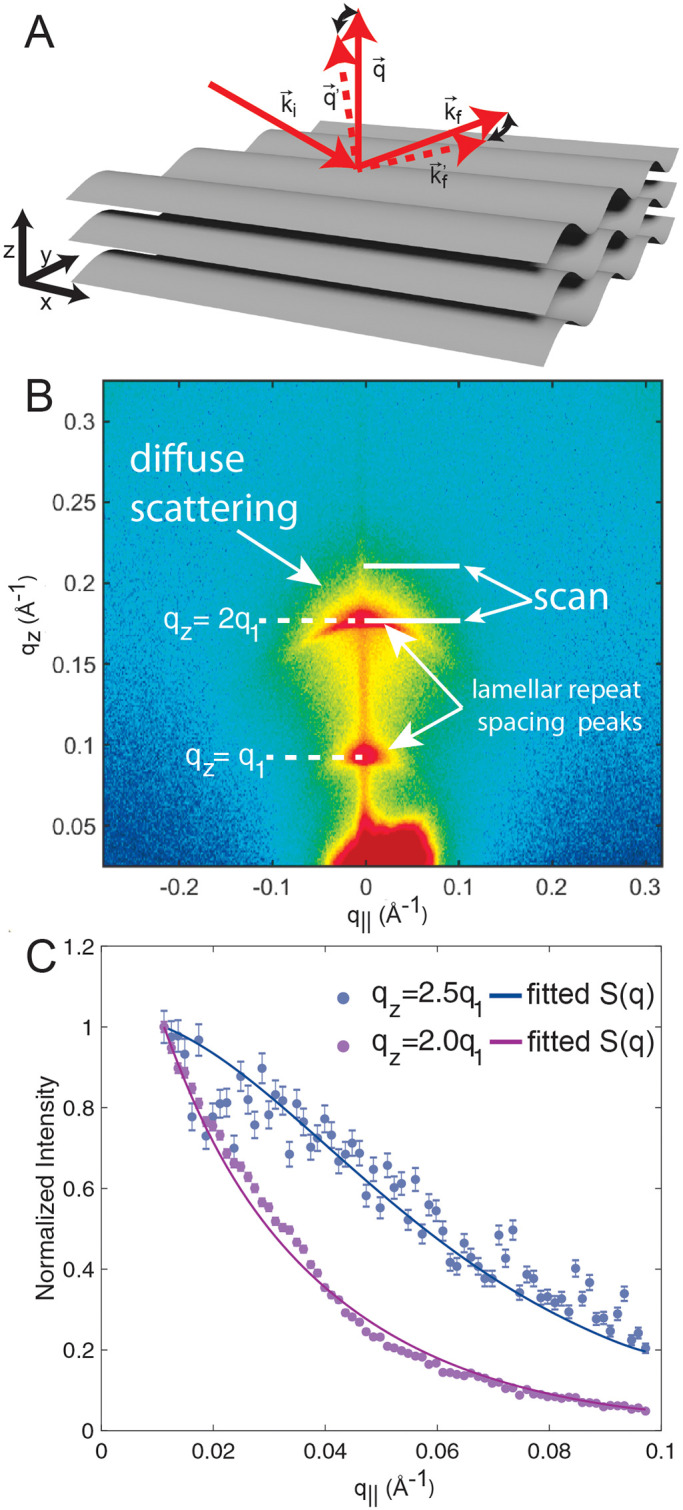
**A** Schematic of a stack of fluctuating membranes and the geometry of specular (*q*_||_ = 0) and off-specular (*q*_||_ ≠ 0) X-ray scattering. **B** *q*-space X-ray intensity map of a solid supported RBC_*cm*_ membrane stack, measured at 37°C. Two orders of lamellar peaks surrounded by diffuse X-ray intensity are visible. The white lines show the locations and ranges of the data used for fits. **C** Off-specular intensities at *q*_*z*_ = 2*q*_1_ and *q*_*z*_ = 2.5*q*_1_, normalized to the respective X-ray intensity at *q*_||_ = 0.01 Å^−1^). Fits of the structure factor *S*(*q*_*z*_, *q*_||_) following Eq (1) are shown as solid lines. Error bars represents the ± standard deviation. Intensity measured in proximity of a lamellar peak is orders of magnitude higher than intensity measured in between lamellar peaks. Since errors in scattering experiments scale with the square root of counted X-ray photons, the relative error is consequently smaller in proximity of a lamellar peak than between lamellar peaks.

This model was fit simultaneously to the intensities at *q*_*z*_ = 2.0*q*_1_ and *q*_*z*_ = 2.5*q*_1_ to obtain values of *κ* and *B* with results shown in Fig 1C. Further details of the numerical calculation of the structure factor in Eq (1) are described in the *Supplementary Material*.

Only two lamellar repeat spacing peaks were detected for solid supported RBC_*cm*_, limiting the analysis of the membrane fluctuations to the low-*q*_*z*_ regime. The observed diffuse X-ray signal is secondarily sensitive to the domain size [16, 34], so the primary fit was repeated for different values of *L*_*r*_ and the fit with the smallest *ξ*^2^ was found for *L*_*r*_ = 500 Å.

The direct application of the XDS method gives values of *κ* ≈2 k_B_T that we believe are too small. The main reason is that molecular tilt was not included in Eq (9) because our experimental setup gave too low signal/noise to provide a meaningful fit to an extra parameter. Inclusion of tilt generally increases *κ* by 25% to 50% as the tilt modulus *K*_*t*_ varies from 90 mN/m to 50 mN/m when *κ* is of order 20 k_B_T [36]. However, our simulations suggest that *K*_*t*_ is only about 4 mN/m for the RBC_*cm*_. In order to estimate how much *κ* of the RBC_*cm*_ would increase if the XDS data were strong enough to fit for *K*_*t*_, we prepared several sets of emulated structure factor data with different values of *κ*_*emu*_, all with the simulated value of *K*_*t*_. We then fit these emulations with the tilt independent XDS analysis program used for actual data to obtain *κ*_*fit*_ values. The emulated data that returned *κ*_*fit*_ closest to the value of 2 k_B_T had a value of *κ*_*emu*_ that was about 6 k_B_T. This is the value shown as the large end of the range in Table 1.

Importantly, the structure factor for the emulated data had very weak peaks for orders three and higher, in agreement with the primary data shown in Fig 1. Those intensities are a product of the structure factor and the form factor squared; the latter could also have extinctions, but invoking extinctions is not necessary to account for the absence of higher order peaks which comes about just from the effect of small values of the elastic moduli on the structure factor.

Our fitting routine was tested on a POPC bilayer. The observed 2-dimensional X-ray scattering pattern is shown in S3A Fig in S1 File and the corresponding diffuse profiles are depicted in S3B Fig in S1 File including fits to the data. The determined bending modulus of (20.8±1) k_B_T is in good agreement with the earlier 20.3 k_B_T [37] and the more recent tilt independent value of 19.2 k_B_T [38], which further supports the use of this analysis for the RBC_*cm*_.

### Neutron spin-echo spectroscopy

Neutron Spin-Echo (NSE) experiments were performed using the NGA-NSE spectrometer at the NIST Center for Neutron Research (NCNR) in Gaithersburg, MD, USA [39]. While XDS measures nearly instantaneous snapshots of the disorder caused by the fluctuations, NSE measures the relaxation rates of those fluctuations which are affected by transport properties like viscosity as well as the static bending modulus. Measurements were performed at *q* = 0.0523 Å^−1^, 0.0664 Å^−1^, 0.0794 Å^−1^ and 0.0959 Å^−1^ using neutron wavelengths of λ = 8 Å and 11 Å, with a wavelength spread *Δλ*/λ≈0.18, providing access to Fourier times ranging from 0.01 ns to 100 ns. Temperature was controlled to 37°C by a recirculation bath within an accuracy of ±1°C. Data were corrected for instrumental resolution and solvent background using the DAVE software package [40].

Eq (12) relates the effective bending modulus $\tilde{\kappa }$ to the customary bending modulus *κ*. The most important step to obtaining the true bending modulus *κ* is to relate *K*_*A*_ to *κ*. The relation

$$\begin{array}{c} K_{A}=48\frac{\kappa }{{\left(2D_{c}\right)}^{2}}, \end{array}$$ (4)

where 2*D*_*c*_ is the thickness of the hydrocarbon region, has been used [23] for NSE experiments on pure lipid bilayers. (We note that [23] incorrectly calls Eq (4) the *polymer brush model*. Instead, the factor 48 assumes uncoupled uniform monolayers, and the polymer brush model would replace 48 by 24.) Assuming Eq (4) and Eq (12), Eq (11) can be written as

$$\begin{array}{c} \Gamma_{nse}^{pb}=0.025{\left(\frac{k_{B}T}{\kappa (1+48{\left(h/2D_{c}\right)}^{2})}\right)}^{1/2}(\frac{k_{B}T}{\eta })q^{3}. \end{array}$$ (5)

It is often assumed that the neutral surface *h* (defined as the location in each monolayer where stretching is decoupled from bending [41]) is close to the boundary *D*_*c*_ of the hydrocarbon chains and the head group, in which case *h*/*D*_*c*_ = 1. Previous studies of the electron density of red blood cell membranes report a head-head group distance (membrane thickness) of 46 Å [32, 42]. Using these results, the thickness of the hydrophobic core is estimated to be 2*D*_*c*_ = 36 Å. Using the uncoupled monolayer model results in a value of *κ* = (15±1.6) k_B_T.

Pan *et al.* [19] pointed out that conventional models, the uncoupled monolayer model in Eq (4), the coupled monolayer model, and the polymer brush model, did not account for *κ* and *K*_*A*_ data as cholesterol was added to lipid bilayers. Evan Evans provided an alternative theory [19] that assumed a stiff region in both uncoupled monolayers with a length of *δ* = 9 Å. This resulted in

$$\begin{array}{c} K_{A}=12\frac{\kappa }{\delta^{2}} \end{array}$$ (6)

Consequently,

$$\begin{array}{c} \Gamma_{nse}^{chol}=0.025{\left(\frac{k_{B}T}{\kappa (1+12{\left(h/\delta \right)}^{2})}\right)}^{1/2}(\frac{k_{B}T}{\eta })q^{3}. \end{array}$$ (7)

Using *h* = *D*_*c*_ and *δ* = 9 Å, the result for this cholesterol model is *κ* = (4.1±0.4) k_B_T when no diffusion correction was made and *κ* = (7±0.4) k_B_T when a diffusion correction was made; this is the range of values we display in Table 1 for our NSE results.

This analysis also makes a direct connection to the area compressibility *K*_*A*_ via Eq (6). Using *κ* = 4 k_B_T gives *K*_*A*_ = 250 mN/m which is close to the values for single component saturated and unsaturated lipid bilayers [11]. In contrast, for the intact RBC *K*_*A*_ = 500 mN/m [43], but this should be the sum of the area compressibility *K*_*A*_ of the RBC_*cm*_ and of the spectrin network because the *K*_*A*_ of a composite membrane is the sum of the two parallel substituents [44]. This implies *K*_*A*_ = 260 mN/m for the spectrin network. If instead, we use our larger *κ* = 7 k_B_T this gives 438 mN/m for the RBC_*cm*_, leaving 62 mN/m for the spectrin network, so either value is reasonable. In contrast, Eq (4) only gives 62 mN/m for the RBC_*cm*_ membrane *K*_*A*_, which is smaller by far than any known lipid bilayer.

It has been previously discussed [45] that vesicle diffusion can contribute a correction factor of exp(−*Dq*^2^*t*) to Eq (10), where the diffusion constant *D* can be estimated from the mean size of the vesicles. While diffusion dominates NSE relaxation for *qR* ≪25, its influence becomes considerably smaller for our mean vesicle radius (*R* = 200 nm) and the experimental *q* range, so its effect is often ignored [23]. We have therefore analyzed our data with and without a diffusion correction factor and thus obtain the range of values of *κ* shown in Table 1.

### Molecular dynamics simulations

MD simulations were performed on a GPU accelerated computer workstation using GROMACS Version 5.1.4. An RBC_*cm*_ model was designed using the CHARMM-GUI membrane-builder (http://charmm-gui.org/) [46, 47] and the Martini force-field 2.2 [47]. The system represents a membrane patch of ≈34 nm × 34 nm with about 2,500 lipid molecules on each leaflet and 37 water molecules per lipid corresponding to a well hydrated state of the membrane.

The lipid composition of the membrane patch was adjusted to match the experimental lipidomic findings of RBC [48]. Each lipid species was mapped to available models in the Martini force-field as described in [49]: First, an error coefficient was calculated for every available model lipid. This error value is composed of an error of saturation *E*_*sat*_ and an error of tail length *E*_*tail*_. *E*_*sat*_ was chosen to be the difference in tail saturation between the model and the experimental lipid. In the same way *E*_*tail*_ was defined as the difference in tail length. For instance, given an experimental Lipid: 18:2–14:1; a corresponding Martini lipid 18:1–16:1 would result in an error value of *E* = *E*_*sat*_+ *E*_*tail*_ = 1 + 2 = 3. The Martini lipid with the smallest error value was then used for each experimental lipid respectively. The cholesterol concentration was taken from [50] who reported that cholesterol accounts for a mole fraction of 50% of the RBC_*cm*_.

The RBC_*cm*_ is known to be asymmetric, with phosphatidylserine (PS) and phosphatidylethanolamine (PE) lipids preferably located on the inner leaflet. This asymmetry between different lipid species was adjusted by using values for the compositional asymmetry published in previous coarse grained plasma membrane simulations [51]. For a given species the simulated lipid population was split among both leaflets to match the relative experimental findings. For instance, phosphatidylcholine (PC) lipids were reported to be split in a ratio of 3:1 between the upper and lower leaflet. Thus from all simulated PC lipids 75% were placed in the upper and 25% were placed in the lower leaflet. Details about the exact lipid composition of each model can be found in the S1 Data. S5 Fig in S1 File visualizes the relative concentrations of lipid species in the membrane model.

Simulations were equilibrated for 80 ns in the NPT ensemble (constant pressure and temperature), and then run for 5 *μ*s. Prior to each simulation run, the system was allowed to equilibrate for simulated 5 ns. The simulation used a 1 fs time step, a short range van der Waal cutoff of 1.1 nm and a potential-shift-verlet coulomb modifier. Periodic boundary conditions were applied to all spacial directions. We note that periodic boundary conditions discretize the wave-vectors and result in a low-Q limit of the accessible fluctuation spectrum *Q*_*min*_ = 2*π*/*L*, where L is the box size.

Neighbor lists were updated in intervals of 20 steps. The temperature coupling was controlled by a *v*-rescale thermostat at a constant pressure of 1 bar using Parrinello-Rahman semi-isotropic weak coupling (*τ* = 12 ps; compressibility *β* = 3 ⋅ 10^−4^ bar^−1^). The fluctuation spectrum of the membrane was determined as detailed in the *Supplementary Material*. The spectrum is governed by a $Q_{\left|\right|}^{-4}$ dependency according to the Helfrich–Canham (HC) theory plus a $Q_{\left|\right|}^{-2}$ dependency due to tilt [52]. We use $\vec{Q}$ with the in-plane component *Q*_||_ to distinguish between the Fourier space of the sample and the scattering vector $\vec{q}$. The bending modulus was determined by fitting the lower *Q*_||_-regime (*Q*_||_<0.2 Å^−1^) to

$$\begin{array}{c} \langle |h\left(Q_{\left|\right|}\right){|}^{2}\rangle =\frac{k_{B}T}{\kappa Q_{\left|\right|}^{4}}+\frac{k_{B}T}{K_{t}Q_{\left|\right|}^{2}} \end{array}$$ (8)

The MD simulations were conducted in the absence of any proteins in order to specifically study the influence of the lipid membrane on the bending modulus. The analysis of the XDS experiment is based on smectic elastic theory and does not include potential protein induced local curvature. Simulating a bilayer in the absence of proteins thus allows a direct comparison between both methods and provides insight into the contribution of the lipid bilayer to the RBC_*cm*_ bending rigidity. The simulated value of *κ* essentially agrees with those from XDS and NSE. This suggests that *κ* can in first order be well approximated by the properties of just the lipid membrane.

Determining the bending modulus in asymmetric membranes is non-trivial due to potentially induced curvature resulting from an uneven area per lipid in both leaflets [53]. Simulations on membrane patches with symmetrized upper and lower leaflet were used to verify the results from the asymmetric simulation. The resulting fluctuation spectra are presented in S4 Fig in S1 File. We find values of 5 k_B_T and 6 k_B_T, respectively which confirms the results that we obtained for the asymmetric membrane.

### Dynamic light scattering

The size distribution of the liposomes was measured by dynamic light scattering (DLS) using a Brookhaven 90Plus particle analyzer running Particle Solutions Software (Version 2.6, Brookhaven Instruments Corporation) with a 659 nm laser and a 90° detection angle. Each measurement was performed at a count rate between 200 and 500 kilocounts/s for 2 min. The scattering signal at the position of the detector fluctuates due to the diffusion of liposomes in the solution. The instrument directly measures the diffusion constant *D* of the liposomes by fitting the cross-correlation function of the time signal measured by the detector. This is related to the particle size via the Stokes-Einstein relation: $D=\frac{k_{B}T}{6\pi \eta r}$, where *η* is the dynamic viscosity of the solution, *k*_*B*_ is the Boltzmann constant, *T* is the sample temperature and *r* is the radius of the LUVs, assumed to be spherical. All measurements were performed at 25°C on 1 ml of sample containing ≈20 mg/ml of RBC liposomes.

### Lipidomics analysis

#### Lipidomics

Samples were resolved as described [54], over an ACQUITY HSS T3 column (2.1 × 150 mm, 1.8 *μ*m particle size (Waters, MA, USA) using an aqueous phase (A) of 25% acetonitrile and 5 mM(mMol/L) ammonium acetate and a mobile phase (B) of 50% isopropanol, 45% acetonitrile and 5 mM ammonium acetate. Samples were eluted from the column using either the solvent gradient: 0–1 min 25% B and 0.3 ml/min; 1–2 min 25–50% B and 0.3 ml/min, 2–8 min 50–90% B and 0.3 ml/min, 8–10 min 90–99% B and 0.3 ml/min, 10–14 min hold at 99% B and 0.3 ml/min, 14–14.1 min 99–25% B and 0.3 ml/min, 14.1–16.9 min hold at 25% B and 0.4 ml/min, 16.9–17 min hold at 25% B and resume flow of 0.3 ml/min. Isocratic elution of 5% B flowed at 250 *μ*l/min and 25°C or a gradient from 0- 5% B over 0.5 min; 5–95% B over 0.6 min, hold at 95% B for 1.65 min; 95–5% B over 0.25 min; hold at 5% B for 2 min, flowed at 450 *μ*l/min and 35°C [55]. The Q Exactive mass spectrometer (Thermo Fisher Scientific, San Jose, CA, USA) was operated independently in positive or negative ion mode, scanning in Full MS mode (2 *μ*scans) from 150 to 1500 m/z at 70,000 resolution, with 4 kV spray voltage, 45 sheath gas, 15 auxiliary gas.

#### MS2 analyses for untargeted lipidomics

For untargeted lipidomics, dd-MS2 was performed at 17,500 resolution, AGC target = 1 ⋅ 10^5^, maximum IT = 50 ms, and stepped NCE of 25, 35 for positive mode, and 20, 24, and 28 for negative mode, as described in Stefanoni *et al.* [48] and applied to similar samples (*i.e.*, stored RBCs) in D’Alessandro *et al.* [56].

#### Quality control and data processing

Calibration was performed prior to analysis using the PierceTM Positive and Negative Ion Calibration Solutions (Thermo Fisher Scientific). Acquired data was then converted from .raw to .mzXML file format using Mass Matrix (Cleveland, OH, USA). Samples were analyzed in randomized order with a technical mixture (generated by mixing 5 *μ*l of all samples tested in this study) injected every 10 runs to qualify instrument performance. This technical mixture was also injected three times per polarity mode and analyzed with the parameters above, except CID fragmentation was included for unknown compound identification (10 ppm error for both positive and negative ion mode searches for intact mass, 50 ppm error tolerance for fragments in MS2 analyses—further details about the database searched below).

#### Metabolite assignment and relative quantitation

Metabolite assignments, isotopologue distributions, and correction for expected natural abundances of deuterium, ^13^C, and ^15^N isotopes were performed using MAVEN (Princeton, NJ, USA) [57], against an in house library of deuterated lipid standards (SPLASH LIPIDOMIX Mass Spec Standard, Avanti Lipids) and in house libraries of 3,000 unlabeled (MSMLS, IROATech, Bolton, MA, USA; IroaTech; product A2574 by ApexBio; standard compounds for central carbon and nitrogen pathways from SIGMA Aldrich, St Louis, MO, USA) and labeled standards (see below for the latter). Untargeted lipidomics analyses were performed with the software LipidSearch (Thermo Fisher, Bremen, Germany). Results from lipidsearch were exported as a library and additional discovery mode analyses were performed with standard workflows using Compound Discoverer 2.1 SP1 (Thermo Fisher Scientific, San Jose, CA). From these analyses, metabolite IDs or unique chemical formulae were determined from high-resolution accurate intact mass, isotopic patterns, identification of eventual adducts (e.g., Na^+^ or K^+^, etc.) and MS2 fragmentation spectra against the KEGG pathway, HMDB, ChEBI, and ChEMBL databases.

## Results

### X-ray diffuse scattering

The geometry of X-ray reflectivity experiments on stacks of membranes is depicted in Fig 1A. The scattering vector $\vec{q}$ is perpendicular to the membrane surface. *q*_*z*_ and *q*_||_ denote the out-of-plane and in-plane component of the scattering vector, respectively. The most intense scattering is specular (*q*_||_ = 0 Å^−1^); as shown in Fig 1B this includes peaks due to the average lamellar repeat distance in the stack of membranes, and it includes the sharp line of reflectivity from the silicon substrate. Only two lamellar repeat spacing peaks are visible for the RBC_*cm*_ samples, indicating a high degree of structural disorder within each membrane. The first order peak was observed at *q*_1_ = 0.084 Å^−1^ corresponding to a membrane *d*-spacing of *d* = 74.8 Å. Most importantly for elastic properties, a cloud of diffuse off-specular scattering was observed. Fig 1C displays the *q*_||_ dependence for *q*_*z*_ = 2*q*_1_ and *q*_*z*_ = 2.5*q*_1_.

Off-specular scattering (*q*_||_ ≠ 0 Å^−1^) is due to thermal fluctuations of membrane undulation modes and compression modes of the stack of membranes. The energy of these fluctuations is given by smectic liquid crystal elasticity theory as [21, 58]

H=∫Ad2r∑n=1N-112(κ(∇||2un)2+B(un+1-un)2∫Ad2r), (9)

where *u*_*n*_ is the locally varying displacement. *κ* is the bending modulus, *B* is the compression modulus, *N* is the number of membranes and *d* is the lamellar spacing between neighboring membranes. Given values of *κ* and *B* this model predicts the structure factor *S*(*q*_*z*_, *q*_||_) [34], which contains the important *q*_||_ dependence of the diffuse scattering intensity. The best values were obtained by varying *κ* and *B* to provide the best fit of this model to the off-specular diffuse intensity. Values of *κ* = 2 k_B_T and *B* = 2 ⋅ 10^−7^ k_B_T/Å^4^ were obtained. This value of *κ* is the smaller of the range of values shown in Table 1. It is known that the true value of *κ* is generally larger because XDS is also affected by the shorter range tilt degree of freedom that adds terms to Eq (9). The larger value of *κ* (*κ* = 6 k_B_T) at the end of the range shown in Table 1 was estimated by including contributions from tilt, as described in Materials & methods. The modulus *B* accounts for interactions between neighboring membranes and it decreases dramatically and exponentially with increasing *d*. The values we obtain are consistent with the range shown for a typical bilayer in Fig 8 in [59]

### Neutron spin echo

**Fig 2:**
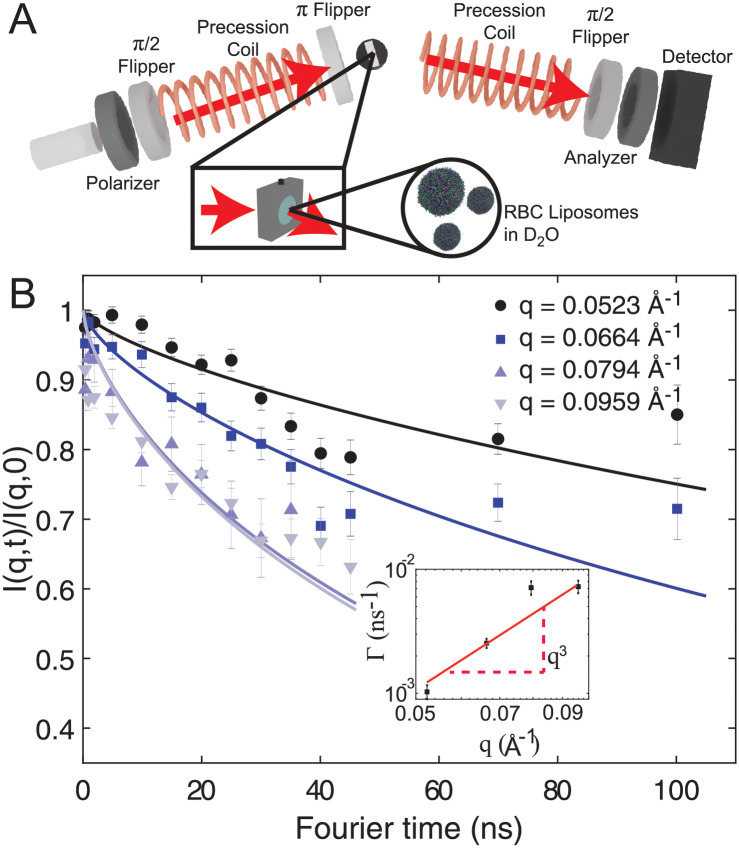
**A.** Experimental setup of the NSE experiment. 6 ml of RBC_*cm*_ liposomes immersed in D_2_O at a concentration of 20 mg/ml were filled in custom made titanium/quartz chambers provided by the NIST Center for Neutron Research (NCNR). **B** Intermediate scattering function *I*(*q*, *t*)/*I*(*q*, 0) at *q* = (0.0523, 0.0664, 0.0794 and 0.0959) Å^−1^. Data were fitted by Eq (5). The inset shows the determined decay constant Γ as function of the scattering vector *q* which were fitted with a *q*^3^ dependency, as predicted by the Zilman-Granek theory. A bending modulus of 4 k_B_T was determined using the cholesterol model. (Error bars represents the ± standard deviation).

Membrane dynamics were measured in unilamellar liposomes by NSE. The precession of the neutron spin in a well-defined magnetic field is used to determine the energy transfer between neutrons and membranes on length scales of ≈10 nm. The basic set up of the experiment is shown in Fig 2A. Nanometer sized RBC_*cm*_ liposomes were immersed in D_2_O and mounted in a custom-made titanium chamber. Details of the experimental setup are described in Materials & methods. The liposome size distribution was measured by DLS giving respective diameters of (199 ± 3) nm (polydispersity index: 0.1 ± 0.01). The DLS results explain the SANS sspectrum that was measured prior to the NSE experiment and is shown in S2 Fig in S1 File. A membrane thickness of 53.8±0.2 Å was determined in good agreement with previously published results [32]. Data for the NSE intermediate scattering function were fitted to the Zilman and Granek [60] theory,

$$\begin{array}{c} I\left(q,t\right)/I\left(q,0\right)=\text{exp}[-{\left(\Gamma t\right)}^{2/3}], \end{array}$$ (10)

as shown by solid lines in Fig 2B. The inset shows the decay constant Γ versus *q*. These values of Γ were then used to obtain the effective bending modulus $\tilde{\kappa }$ using

$$\begin{array}{c} \Gamma \left(q\right)=0.025{\left(\frac{k_{B}T}{\tilde{\kappa }}\right)}^{1/2}(\frac{k_{B}T}{\eta })q^{3}, \end{array}$$ (11)

where *k*_*B*_*T* is thermal energy and *η* is the solvent viscosity.

The theory of Watson and Brown [61] relates the effective bending modulus $\tilde{\kappa }$ to the customary bending modulus *κ* by the formula

$$\begin{array}{c} \tilde{\kappa }=\kappa +h^{2}K_{A}, \end{array}$$ (12)

that also involves the area compressibility modulus *K*_*A*_ and the distance *h* of the neutral surface of each monolayer from the bilayer midplane. To obtain *κ* it is necessary to eliminate *K*_*A*_. As detailed in Materials & methods, the range of values of *κ* = 4 k_B_T to *κ* = 7 k_B_T listed in Table 1 is the result of a model that is appropriate for bilayers with a high concentration of cholesterol [62, 63]. The high end of this range also included a correction factor for diffusion of the unilamellar liposomes in Eq (10) whereas the low end of this range did not attempt to compensate for liposome diffusion.

### Molecular dynamics simulations

**Fig 3:**
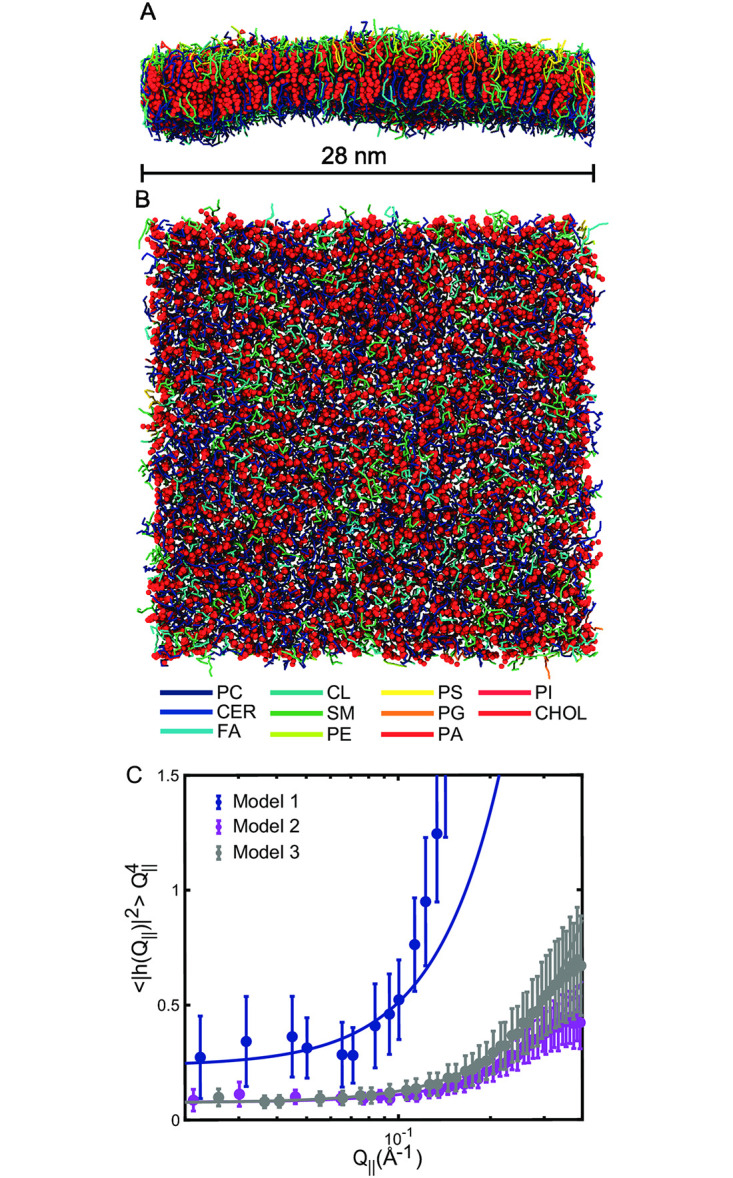
Three-dimensional renderings of the undulation simulation after 5 *μ*s (side-view A and top-view B). Lipid molecules are represented by rods representing intra-molecular bonds. Each lipid species (Phosphatidylcholine, PC; Ceramide, CER; Monoglucosyl lipids, MG; Diacylglycerol lipids, DG; Fatty acids, FA; Sphingomyelin, SM; Phosphatidylethanolamine, PE; Phosphatidylserine, PS; Phosphatidylglycerol, PG; Phosphatidic acid, PA; Phosphatidylinositol, PI) are represented by different colors indicated in the legend. Cholesterol (CHOL) is symbolized by red spheres. **C** The fluctuation spectrum determined from the undulation simulation averaged over the last 4 *μ*s for all three models. Fits of Eq (8) to the data are depicted as red solid line. The fit range was *Q*<0.2 Å^−1^. (Error bars represents the ± standard deviation).

We used results from mass spectrometry on extracted lipids from native RBCs [48] for the lipid composition of the RBC_*cm*_. The cholesterol concentration was not determined but taken from [50] reporting a cholesterol to lipid ratio of ≈1. Three membrane models containing ≈4,000 molecules forming a patch of ≈30 nm × 30 nm were created. For the first model, the determined membrane composition was recreated in coarse grained MD simulations by mapping experimental lipids to the molecules available in the MARTINI force-field. This model will be referred to as *Model 1*. In a second model, we removed any lipid molecule with two or more unsaturated bonds per tail (*Model 2*). Cholesterol accounted for a mole fraction of 50% of both membrane models. In *Model 3*, we removed all cholesterol from *Model 1*. Details about the mapping process can be found in Materials & methods and the model composition is listed in S1 Data. Fig 3A and 3B show a 3-dimensional rendering of *Model 1* (side- and top-view).

The undulation spectrum was determined and is shown in Fig 3C. The spectrum has a $Q_{\left|\right|}^{4}$ dependency in the low-*Q*_||_ regime (*Q*_||_<0.2 Å^−1^) in good agreement with the Helfrich–Canham (HC) theory (Eq (8)). This theory models the membrane as an elastic sheet and is only valid for length scales much larger than the membrane thickness, *i.e.* small *Q*_||_ [64, 65]. The spectrum consequently differs from the $Q_{\left|\right|}^{4}$ dependency for *Q*_||_>0.2 Å^−1^ due to molecular tilt that is characterized by the tilt modulus *K*_*t*_ [52]. Fits of Eq (8) for values of *Q*_||_<0.2 Å^−1^ are displayed as a red solid line from which the bending modulus and tilt modulus were determined to be *κ* = (4.2±0.8) k_B_T and *K*_*t*_ = (3.63±1) mN/m. The bending rigidity and the membrane’s tilt modulus were both found to increase in *Model 2* and values of *κ* = (13±0.6) k_B_T and *K*_*t*_ = (30.4±1.5) mN/m were determined. A similar increase was also observed in Model 3 and values of *κ* = (13±0.2) k_B_T and *K*_*t*_ = (20.2±0.3) mN/m were determined.

## Discussion

We have measured the bending modulus *κ* of the cytoplasmic RBC membrane in the absence of the spectrin network. Using our prepration protocol of sonication with subsequent centrifugation of RBC liposomes, spectrin filaments could no longer be detected by fluorescent microscopy [32]. In addition, the *d*-spacing in X-ray diffraction experiments together with electron density profiles are inconsistent with the presence of spectrin structures between membranes in the solid supported stack [32, 42, 66]. We now discuss how these results relate to the properties of the intact RBC shell.

The bending rigidity of intact red blood cells has been reported many times, as shown in Table 1. Interestingly, one of the the smallest values, *κ* = 5 k_B_T [7], came from the same lab as one of the largest values 97 k_B_T [8] that was subsequently reported. Likewise, the value of 20 k_B_T [10] was later changed to 7 k_B_T [9]. The disparate experimental results have been appropriately described as puzzling [8, 13]. It was suggested that these apparent controversial results can be explained by the complex interplay between the membrane bilayer and the spectrin network [8].

Correlation of the magnitude of *κ* with the length scale of the experiments has been suggested [13, 67], and one would associate a crossover length scale with the 80 nm mesh of the spectrin network. At length scales substantially greater than that, the composite RBC shell is homogeneous and would be characterized by a bending modulus for both the cytoplasmic membrane and the spectrin network. Measurements on the length scale of the whole cell, such as buckling in an aspiration pipette experiment (43 k_B_T) [4] and deformations induced by optical tweezers (*κ* = 67 k_B_T [12]) would provide these values. For length scales smaller than the crossover length scale, most of the bending would be situated in the cytoplasmic membrane between the ribs of the spectrin network and *κ* values from measurements on those length scales would approach those of just the cytoplasmic membrane, which is likely to be homogeneous down to a length scale of 10 nm.

However, our data do not support the preceding scenario. One may be inclined to explain the large disparity in Table 1 by length-scale effects of the bending modulus *κ*. Indeed, the smaller values by Brochard *et al.* (*κ* = 3 to 9 k_B_T [6]), and Park *et al.* (*κ* = 7 k_B_T [9]), Zilker *et al.* (*κ* = 5 k_B_T [7]) are from measurements with small length scales of the order of the wavelength 400 nm of the optical methods employed. However, it is important to appreciate that this is not smaller than the spectrin network length scale of 80 nm, so crossover to the value of *κ* for the cytoplasmic membrane would only be expected to have just begun. At even smaller length scales one would further expect complete crossover to a still smaller value of *κ* which would then be just that of the cytoplasmic membrane. Contrarily, our values for the cytoplasmic membrane are roughly equal to the small values obtained at the optical length scales [6, 7, 9, 31]. We therefore suggest that there is no length scale dependence in *κ*. This implies that there is no contribution of the spectrin network to the RBC bending modulus, in agreement with [14].

Whatever one concludes about the bending modulus for the RBC shell, our methods obtain values of *κ* for the cytoplasmic RBC_*cm*_ in the range of 4 k_B_T to 6 k_B_T. Even though this is a rather large uncertainty range, it is still significant in that the bending modulus of the RBC_*cm*_ is relatively small compared to most pure lipid bilayers, such as POPC, for which *κ* is about 20 k_B_T [17]; this is also the value that we obtained by our analysis of POPC-XDS data in this study to confirm the validity of our implementation of the XDS method (included in the *Supplementary Material*). This may well be attributed to the large lipid diversity in this biological membrane, which is similar to what occurs in HIV mimic membranes [68]. A particularly interesting observation in this context is the increase in both the bending rigidity and tilt modulus in the simulation of *Model 2* for which the polyunsaturated lipids had been removed from the *Model 1* RBC_*cm*_ mimic. This suggests that the softness may be partially explained by the presence of lipids with higher degrees of tail unsaturation within the RBC_*cm*_. It is especially interesting that *κ* of the RBC_*cm*_ is so small when it has 50% cholesterol which is of often found to stiffen bilayers composed of pure lipids. However, it has been shown that the stiffening effect of cholesterol decreases with increasing unsaturation [19] and vanishes already in DOPC and diC22:1PC that have just one unsaturated double bond in each chain. Extrapolation would then suggest that cholesterol might even decrease the bending modulus of membranes with a significant concentration of lipids with multiple double bonds. We indeed observed this in *Model 3*, where we removed cholesterol from the simulation. Importantly, this model still contained polyunsaturated lipid molecules. This points towards a softening mechanism that is driven by the interaction between cholesterol and polyunsaturated lipids.

The nanoscopic regime is most relevant for cellular processes which take place between the ribs of the spectrin network. Especially the passive transport of small molecules is intrinsically related to membrane properties on small length scales [69, 70]. Of course, red blood cells are required to efficiently exchange oxygen and carbon dioxide across the membrane. One may speculate that such permeability is enhanced in a softer membrane, and a standard measure of softness is having a smaller bending modulus. As such, a smaller bending modulus of the RBC_*cm*_ would generally indicate physiological advantage.

Another possible advantage of a small bending modulus of the cytoplasmic membrane might be that it reduces the energy cost for the process of squeezing the RBC through small capillaries. This hypothesis is based on the possibility that such mechanical processes might require local area changes in the cytoplasmic membrane. Such changes could be slaved to changes in the local area of the spectrin network if the latter changes were required. (Even if the spectrin network is rigid with respect to local area changes, changes in its local curvature would necessarily change the local area of the attached cytoplasmic membrane.) It is usual to think of the free energy for area change in terms of the area compressibility modulus *K*_*A*_ which is a fairly stiff modulus, typically 250 mN/m. This modulus is associated with area changes per molecule in a flat membrane and the work done to change the molecular packing. However, in the flaccid, low surface tension regime, the membrane has thermally induced undulations that make the cell’s projected area smaller than the local area [71]. Small increases in the tension pulls out these undulations, resulting in an increase in projected area that corresponds to a much smaller apparent *K*_*A*_ than the one usually reported. Indeed, such an apparent *K*_*A*_ of only 15 *μ*N/m has been reported for the RBC [9]. This means that there is a regime of area strain that costs very little energy. How far the area can change in this low cost regime varies nearly inversely with the bending modulus *κ*. This regime has been measured to extend up to an area increase of about 2% for a lipid bilayer with *κ* = 10 k_B_T [71]. The smaller *κ* of the RBC_*cm*_ thus increases the low-cost regime and would therefore provide a greater range of mechanical flexibility that might be advantageous for blood flow.

## Conclusion

In summary, we have studied the bending of red blood cell membranes by combining X-ray diffuse scattering, neutron spin echo spectrometry and Molecular Dynamics simulations. We determine values for *κ* for the cytoplasmic component of the RBC between 4 k_B_T to 6 k_B_T, which is rather softer than most single component lipid bilayers. This leads us to suggest that nature has designed the RBC to be soft for regions involved in the permeability of gas molecules and also to accommodate possible local area changes.

## Supporting information

S1 File Supplementary Material to the manuscript. (PDF)

S2 File (ZIP)

S1 Data Membrane composition of the performed MD simulations. (XLSX)
