# Supplementary material for: The bending rigidity of the red blood cell cytoplasmic membrane
Source: PLoS One. 2022 Aug 1;17(8):e0269619. doi: 10.1371/journal.pone.0269619 (PMC9342732; doi:10.1371/journal.pone.0269619)
Supplement: S1 File — (PDF) [file pone.0269619.s001.pdf]

Supporting Information to: The Bending Rigidity of the Red Blood Cell Cytoplasmic Membrane

Sebastian Himbert[1,2], Angelo D'Alessandro[3,4], Syed M. Qadri[5], Michael J. Majcher[6], Todd Hoare[6] William P. Sheffield[7,8], Michihiro Nagao[9,10,11], John F. Nagle[12,‡], Maikel C. Rheinstädter[1,2*]

1 Department of Physics and Astronomy, McMaster University, Hamilton, ON, Canada
2 Origins Institute, McMaster University, Hamilton, ON, Canada
3 Department of Pathology and Cell Biology, Columbia University Vagelos College of Physicians and Surgeons and New York-Presbyterian Hospital, New York, New York, USA
4 University of Colorado Denver-Anschutz Medical Campus, Aurora, Colorado, USA.
5 Faculty of Health Sciences, Ontario Tech University, Oshawa, ON, Canada
6 Department of Chemical Engineering, McMaster University, Hamilton, ON L8S 4M1, Canada
7 Department of Pathology and Molecular Medicine, McMaster University, Hamilton, ON, Canada
8 Centre for Innovation, Canadian Blood Services, Hamilton, ON, Canada
9 Center for Neutron Research, National Institute of Standards and Technology, Gaithersburg, MD 20899, USA
10 Department of Materials Science and Engineering, University of Maryland, College Park, MD 20742, USA
11 Department of Physics and Astronomy, University of Delaware, Newark, DE 19716, USA
12 Department of Physics, Carnegie Mellon University, Pittsburgh, PA 15213, USA

‡ nagle@cmu.edu * Department of Physics and Astronomy, McMaster University, ABB-241, 1280 Main Street West, Hamilton, Ontario L8S 4M1, Canada; Phone: +1-(905)-525-9140-23134, Fax: +1-(905)-546-1252, E-mail:rheinstadter@mcmaster.ca

X-ray diffraction experiments

X-ray diffraction measurements were performed using CuKα X-rays (λ=1.5418 Å) generated by a RIGAKU SmartLab rotating anode instrument operated at 9 kW. The focusing multi-layer optics provided a high intensity circular beam with a diameter of ≈200 μm and an angular divergence of 0.008 rad with monochromatic X-ray intensities of 10^8 counts/mm^2·s. The instrument is equipped with a Rigaku HyPix-3000 2-dimensional semiconductor detector with an array of (n, m) pixels of size 100 μm^2. We note that this detector counts single photons in every pixel in contrast to widely used CCD based instruments. The geometry of the instrument is sketched in Fig. S1.

Both source and detector were moved on spherical coordinates around the stationary horizontal sample allowing to control the incident angle θ. As θ was varied, the scattering intensity measured by the detector was read only for the pixel row matching the specular condition in its center as indicated by the blue highlighted pixels in Fig. S1. These intensities at q_z were then recorded at the corresponding q_z value in the data set shown in Fig. 1 B in the main text, which was used for analysis.

The sample holder was a sealed chamber with two double walled kapton windows on either side. A basin at the bottom below the sample was filled with aqueous solution and the humidity inside the chamber was controlled by the salinity of this solution. Ultra pure water was used for experiments performed near 100 % RH and the d-spacing of a POPC sample was as large as is obtained for fully hydrated unoriented multilamellar vesicles in bulk water. The RBC samples at 100 % RH had an unbounded d-spacing due to the presence of charged lipids, so the humidity was tuned to 99 % RH by using a 40 mg/ml K_2SO_4 solution to obtain a finite lamellar spacing d necessary for analysis of the moduli [1]. The temperature inside the chamber was 37 °C.

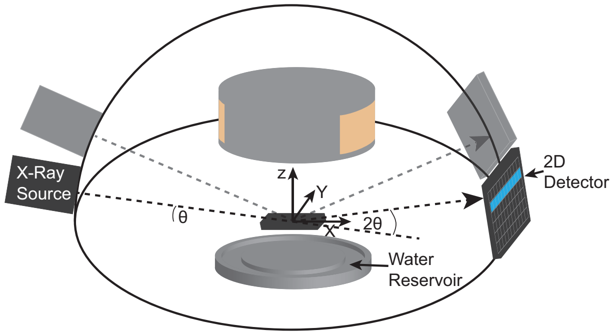

Figure S1. Schematic illustration of the instrumental setup. The X-ray source and the 2-dimensional detector, mounted on movable arms, were simultaneously rotated by $\pm\theta$ relative to the fixed horizontal sample. The detector recorded only the pixel row indicated by the blue highlighted pixels whose center was at the specular relative to the incident angle θ. The double walled aluminum chamber consisted of a lid with two double walled kapton windows on either side. A solution reservoir beneath the sample provided the desired relative humidity inside the tightly sealed chamber.

We emphasize that the sample remained horizontal throughout the measurement. This differs from the earlier protocol [3] in which the sample was rocked while the synchrotron source and the detector remained fixed. In that protocol the intensity at each pixel came from a trajectory in q space whereas each pixel in the present protocol received intensity from only one point in q space; this simplified the XDS analysis which was rewritten for this setup [2], following instructions from [1]. While simpler, calculating the structure factor in Eq. (1) was still computationally challenging. First a table of $\delta u_n(r)$ was calculated numerically for $n \leq 30$ and $r \leq 1000$ Å using logarithmic steps in r. For $n > 30$ and 1000 Å$< r < 10^6$ Å the approximation proposed by Callié

was used as in [1]:

$$\delta u_n(r) = \frac{4\eta_c}{q_1^2}\left[\gamma \ln\left(\frac{r}{\xi}\right) + 0.5E_1\left(\frac{r^2}{4n\xi^2}\right)\right], \qquad \text{(Eq. (S1))}$$

where γ is Euler's constant and E_1 is the exponential integral. The same way, tables were calculated for $H_r(r, L_r, \sigma_r)$ and $H_z(z, L_z, \sigma_z)$. Then the summation in Eq. (1) ($n \leq 1000$) was calculated from these predetermined tables using a GPU accelerated algorithm. The Hankel transformation in Eq. (1) was then calculated using the Simpson-rule allowing this step to be accelerated through the GPU.
Levenberg-Marquardt least square fitting (GNU Scientific library: *gsl_multifit_nlin*) was then used to obtain the κ and B values for which $S(q)$ best fit the data, necessarily allowing each value of q_z a different normalization factor related to the electron density profile of the membrane. Data used were the measured X-ray intensity slices at $q_z = 2q_1$ and $q_z = 2.5q_1$. The program is available upon request from the authors of this paper. Interestingly, essentially the same values of κ and B were obtained by fitting all q_z slices between $q_z = 2q_1$ and $q_z = 2.5q_1$ using the program for the original experimental protocol. [3]. Also, for the control POPC, the range of q_z for fitting was moved to $q_z = 3q_1$ and above, as in previous studies [3], because the diffuse scattering is more robust compared to the specular due to the larger bending modulus. Again, both methods of fitting gave essentially the same values of the moduli.

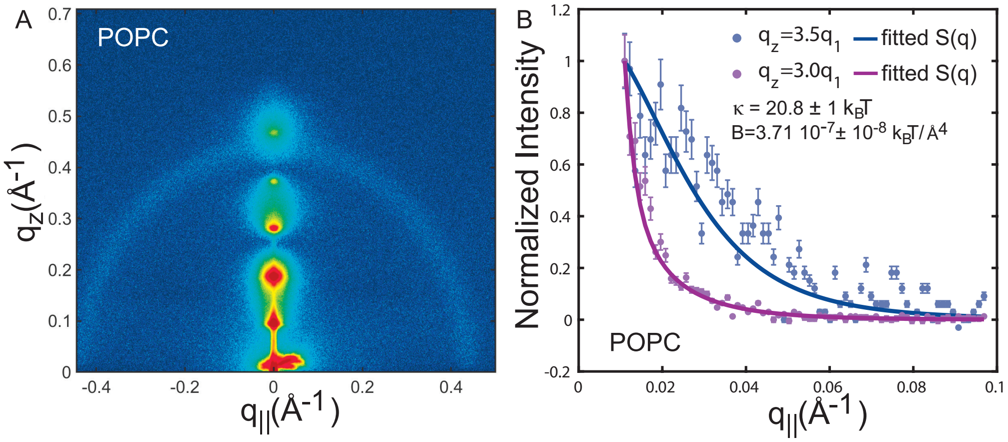

Figure S2. **A** 2-dimensional intensity map of a POPC bilayer measured at 100 % relative humidity. **B** Diffuse profile extracted at q_z=3q_1 and 3.5q_1. Fits of $S(q)$ (Eq. (1)) are shown as solid lines. Error bars represents the ± standard deviation. Intensity measured in proximity of a lamellar peak is orders of magnitude higher than intensity measured in between lamellar peaks. Since errors in scattering experiments scale with the square root of counted X-ray photons, the relative error is consequently smaller in proximity of a lamellar peak than between lamellar peaks.

Small angle neutron scattering (SANS)

SANS experiments were conducted using the 30 m SANS NGB30 at the NIST Center for Neutron Research (NCNR, Gaithersburg, MD). Sample-to-detector distances of 1 m, 4 m and 13 m with a neutron wavelength of 6 Å were used to measure a q range between 0.003 Å^{-1} and 0.4 Å^{-1}. RBC liposomes in D_2O were loaded into NCNR's custom quartz sample holders (diameter 19 mm and thickness 1 mm, corresponding to a volume of ≈800 μl per sample). The low q range data were acquired by counting for 120 min

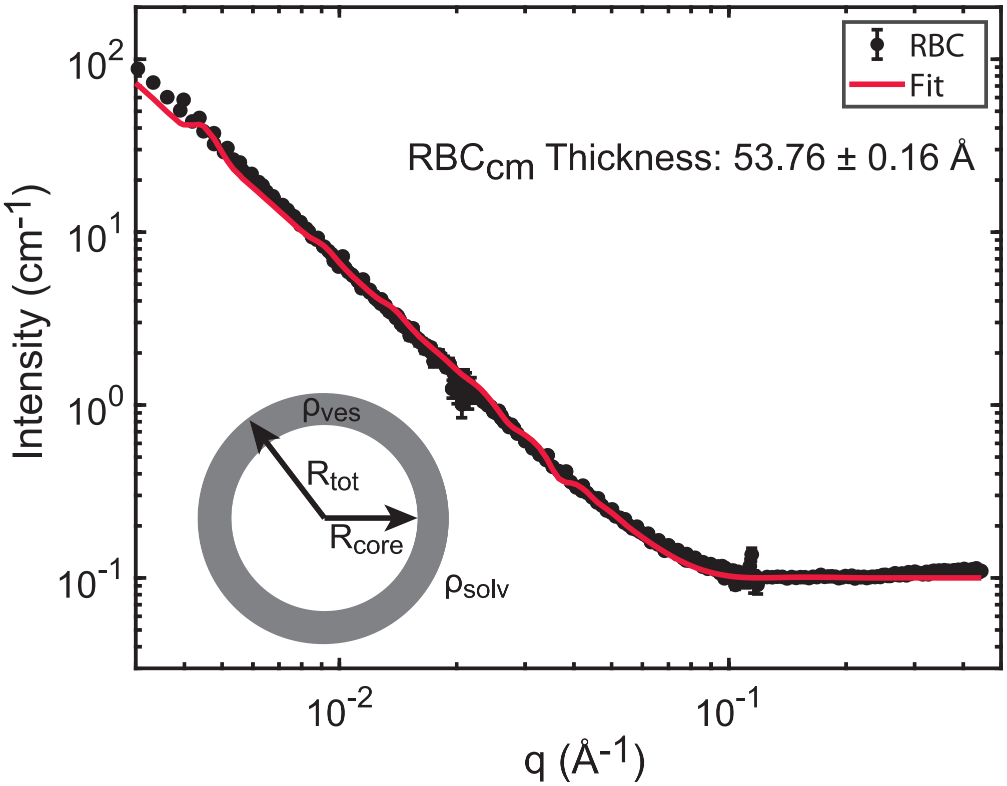

Figure S3. SANS curves recorded on RBC liposomes in D_2O. Data were fit to a vesicle model (Eq. (S2)) and the fit is shown as solid red line. (Error bars represents the ± standard deviation)

using the 13 m configuration, the medium q range data were acquired by counting for 10 min using the 4 m distance, and the high q range data were acquired for 5 min using the 1 m detection distance. The three ranges were reduced and merged using Igor Pro Version 6.37 and macros provided by the NIST-NCNR. The data were fit to the vesicle model [7] using SASVIEW version 5.0.2. The structure factor in this model is given as:

$$S(q) = \frac{\varphi}{V_{shell}}\left[\frac{3V_{core}(\rho_{solvent}-\rho_{shell})J_1(qR_{core})}{qR_{core}} + \frac{3V_{tot}(\rho_{shell}-\rho_{solvent})J_1(qR_{tot})}{qR_{tot}}\right]^2 + \text{Background}, \quad \text{(Eq. (S2))}$$

where φ is the volume fraction, V_{shell} is the membrane volume, V_{core} is the volume of the vescile core and $V_{total} = V_{shell} + V_{core}$. J_1 is the first order bessel function. R_{core} and R_{tot} referring to the core radius and total vesicle radius respectively.

Molecular Dynamics simulations

MD simulations were performed on a GPU accelerated computer using GROMACS Version 5.1.4. The device is equipped with a 40 Core central processing unit (CPU, Intel(R) Xeon(R) CPU E5-2630 v4 @ 2.20GHz), 130 GB random-access memory (RAM) and three graphic processing units (GPU, 2 × NVIDIA 1080 TDI + 1 × GeForce GT 730).

The fluctuation spectrum was determined as follows: First the upper and lower leaflet was indext using the *spliptleaflets* program. The position of C1 Beads from

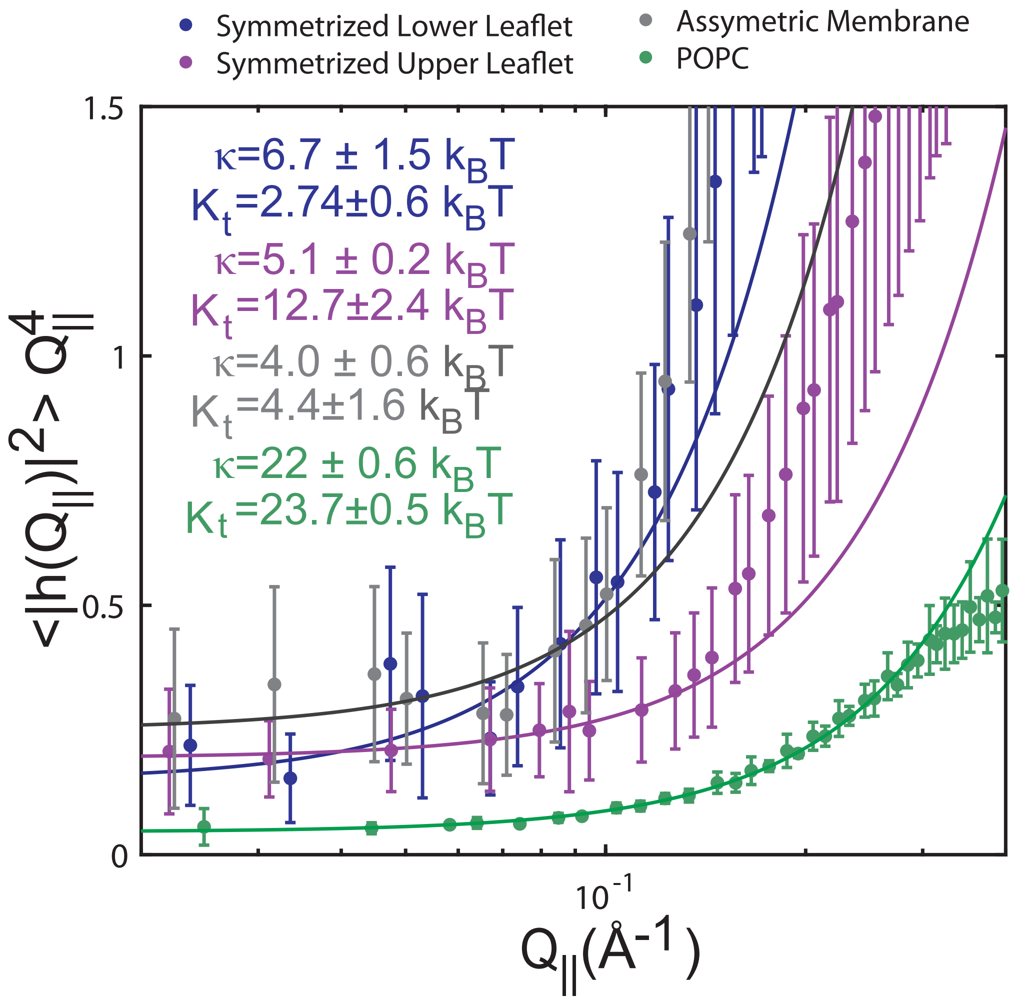

Figure S4. Fluctuation spectra determined from simulations of a POPC bilayer and symmetrized versions of the asymmetric membrane patch. Bending moduli of κ=(19.7±2) k_BT, κ=(4.1±1) k_BT and κ=(3.1±0.8) k_BT were determined for POPC, and RBC_{cm} membranes with a symmetric upper and lower leaflet respectively. (Error bars represents the ± standard deviation)

DPGG, OPGG, FPGG, and DFGG, as well as GL1 beads from FPMG, OPMG, DPMG, were exported between 200 ns and 5 μs in steps of 4 ns for each leaflet separately together with the position of the PO4 beads from the remaining lipid molecules. The Z position from all atoms was interpolated using a 2-dimensional cubic interpolation provided by the MATLAB built-in *griddata* function for both leaflets respectively. The membrane undulation-profile was then determined by calculating the average undulation of the upper and lower leaflet. The 2-dimensional spectrum was then determined using built-in MATLAB function. The scaling of the spectrum was verified using the program provided by the authors of [6] and a simulation of a POPC bilayer. A value of κ =(19±2) k_BT was determined, as shown in Fig. S4 in the *Supplementary Material*, in good agreement with previously published results.

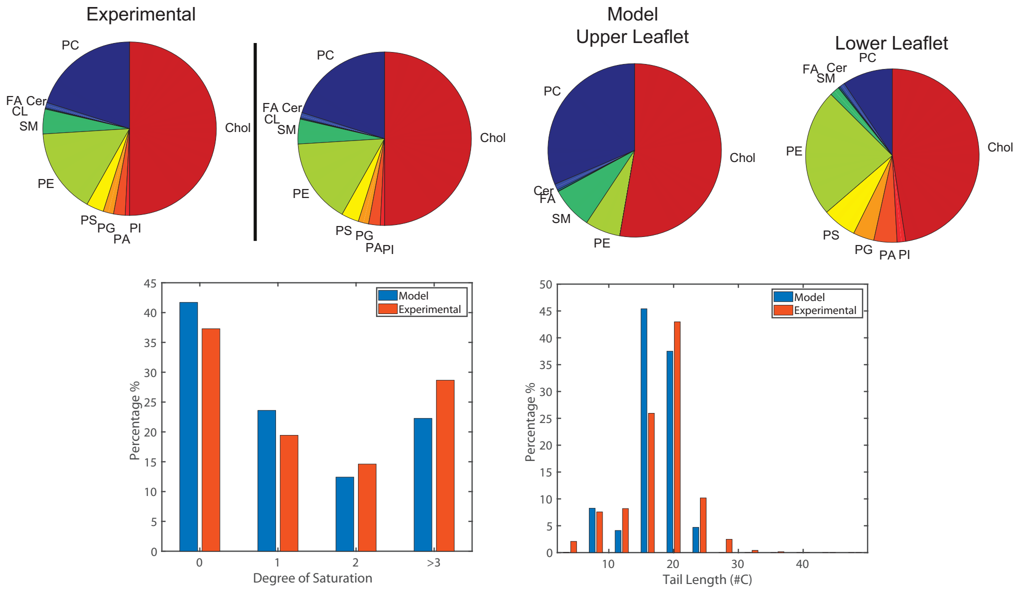

Figure S5. **A** Experimentally determined composition of red blood cell membrane as reported by [4]. **B** Lipidomics of the coarse grained MD simulation model. The asymmetry of the membrane was created by distributing lipids between both leaflets according to experimental findings by [5]. **C** Comparison of the degree of tail saturation in the experimental and model membrane. **D** Comparison between the lipid tail length of the experimental and model membrane.

Disclaimer

This research was approved by the Hamilton Integrated Research Ethics Board (HIREB) under approval number 1354-T. Informed consent was obtained from all blood donors. The authors confirm that all methods were performed in accordance with the relevant guidelines and regulations. This research used the neutron research facilities of the National Institute of Standards and Technology, U.S. Department of Commerce. Access to NGA-NSE and the NGB30-SANS was provided by the Center for High Resolution Neutron Scattering, a partnership between the National Institute of Standards and Technology and the National Science Foundation under Agreement No. DMR-2010792. The funders had no role in study design, data collection and analysis, decision to publish, or preparation of the manuscript. Certain trade names and company products are identified in order to specify adequately the experimental procedure. In no case does such identification imply recommendation or endorsement by the National Institute of Standards and Technology (NIST), nor does it imply that the products are necessarily the best for the purpose.

References

1. Lyatskaya Y, Liu Y, Tristram-Nagle S, Katsaras J, Nagle JF. Method for obtaining structure and interactions from oriented lipid bilayers. Physical Review E. 2000;63:011907.

2. Himbert S. Biophysics of Blood Membranes. Doctoral Dissertation, 2021, McMaster University, http://hdl.handle.net/11375/26995.

3. Liu Y, Nagle JF. Diffuse scattering provides material parameters and electron density profiles of biomembranes. Physical Review E. 2004;69(4):040901.

4. Stefanoni D, Shin HKH, Baek JH, Champagne DP, Nemkov T, Thomas T, et al. Red blood cell metabolism in Rhesus macaques and humans: comparative biology of blood storage. Haematologica. 2020;105(8):2174.

5. Dodge JT, Phillips GB. Composition of phospholipids and of phospholipid fatty acids and aldehydes in human red cells. Journal of Lipid Research. 1967;8(6):667–675.

6. Fowler PW, Hélie J, Duncan A, Chavent M, Koldsø H, Sansom MS. Membrane stiffness is modified by integral membrane proteins. Soft Matter. 2016;12(37):7792–7803.

7. Guinier A, Fournet G, Yudowitch KL. Small-angle scattering of X-rays. Wiley New York; 1955.
